# Soil organic carbon stocks after ten years of reduced tillage, compost and mulch application in temperate organic agriculture

**DOI:** 10.1038/s41598-026-42050-9

**Published:** 2026-03-05

**Authors:** Wiebke Niether, Simeon Leisch-Waskönig, Maria R. Finckh, Stephan Martin Junge, Carolina Bilibio, Stephan Peth, Jan Henrik Schmidt, Juliet Wanjiku Kamau, Andreas Gattinger

**Affiliations:** 1 Organic Farming with Focus on Sustainable Soil Use, Justus-Liebig-University of Giessen, Karl-Gloeckner- Str. 21 C, 35394 Giessen, Germany; 2 Ecological Plant Protection, University of Kassel, Nordbahnhofstrasse 1a, 37213 Witzenhausen, Germany; 3 Soil Science, University of Kassel, Nordbahnhofstrasse 1a, 37213 Witzenhausen, Germany; 4 Institute of Earth System Sciences, Leibniz-University-Hannover, Herrenhäuser Strasse 2, 30419 Hannover, Germany; 5 Institute for Epidemiology and Pathogen Diagnostics, Julius Kühn Institute (JKI) - Federal Research Centre for Cultivated Plants, Messeweg 11/12, 38104 Braunschweig, Germany; 6 Center for Development Research (ZEF), University of Bonn, Genscherallee 3, 53113 Bonn, Germany; 7 Institute of Organic Agriculture (FiBL), Kasseler Strasse 1a, 60486 Frankfurt, Germany

**Keywords:** Regenerative agriculture, SOC, Soil quality, Deep C storage, Environmental impact, Agroecology

## Abstract

Increasing soil organic carbon (SOC) aims to increase and maintain soil quality for sustainable crop production and to achieve carbon removal targets. Agronomic practices are therefore needed to reduce carbon losses and increase SOC stocks, especially in deep soil layers, which promote long-term storage. Regenerative agriculture is an approach aimed at increasing soil quality for sustainable production and therefore should be suitable for achieving the required goals under organic farming conditions. We analysed SOC content down to a depth of 1 m and calculated the SOC stock based on bulk density after ten years of regenerative farming practices, i.e., reduced tillage, dead organic mulch, and high-quality yard waste compost application, in two organic field trials set up one year apart in Central Germany and we calculated the carbon (C) and nitrogen (N) input to the soil from organic amendments and from main crops and cover crops by applying C allocation factors for crop residues, roots, and rhizodeposition. C derived from crops was the main carbon input source over ten years. Increasing C input promoted an increase in the cumulative SOC stock down to 1 m. We observed greater SOC stocks dominated by topsoil changes with reduced tillage and compost application and with the combination of all practices (+ 16%) than in the control with conventional ploughing and no external carbon input while none of the farming practices affected the subsoil SOC stock. Mulch application had no effect at all on SOC stocks. Crop biomass contributed most C input. Farming practices, especially the combination of reduced tillage and compost application, enhanced topsoil SOC stocks and N but not subsoil C storage. Other farming practices and crop rotation adjustments must be identified to increase crop production as well as subsoil SOC stocks and promoting long-term C storage, e.g., by fostering deep-rooting crops and cover crops.

**Supplementary Information:**

The online version contains supplementary material available at 10.1038/s41598-026-42050-9.

## Introduction

 Globally, soils are the greatest terrestrial carbon (C) storage with additional storage potential, making them a sink for atmospheric CO_2_ with high potential for climate change mitigation^1^ and achieving greenhouse gas emission reduction targets.

Additionally, soil organic carbon (SOC) also contributes to soil functioning and quality^2^, e.g., through improved soil structure^3^ and water and nutrient storage, leading to increased agricultural productivity and farmers can effectively implement management changes and agricultural practices to increase carbon capture and retention in the soil^4^.

While topsoil has the greatest C storage capacity, it is also prone to faster turnover driven by management, including land use changes, agronomic practices, and climate variability^5–7^.

Subsoils, defined as the soil below a typical management depth, e.g., below depths of 30 cm, often have high clay content and bulk density, as well as limited disturbance, resulting in low aeration and slow mineralization processes^8^ making them suitable for effective and long-term deep C storage^9^. Although most management effects on SOC occur in the topsoil, up to 20% of SOC changes have also been observed below a depth of 30 cm^4^. Agronomic practices must therefore be identified that improve C sequestration and (long-term) storage in the subsoil^8^ while reducing side effects like C leakage and supporting co-benefits, such as the promotion of biodiversity and adaptation to climate change^10^.

In the context of SOC increase, regenerative agriculture is increasingly gaining interest in science, practice, and policies^11^, as it aims to address several current crises in agriculture and food systems^12^. Key targets include restoring degraded land and improving soil quality for sustainable food production and beyond. Agronomic practices such as the use of organic amendments, permanent soil cover, crop diversity, including perennials and legumes, and reduced tillage are promoted to improve the physical, chemical, and biological characteristics of soils^13^. Soil quality, as a principle of regenerative agriculture, is often indicated by SOC and soil nitrogen (N)^2^.

The aim of this study was to measure the effect of farming practices that are typically related to regenerative and organic agriculture – reduced tillage, compost and mulch application - on soil quality indicators such as SOC and N, and the SOC stock in particular. Farming practices were combined over the years of the crop rotation to assess their impact on productivity and to evaluate their deep C storage potential. To avoid potential leakage effects, the biomass (C and N) for organic amendments should (hypothetically) be produced within the farm system boundaries^14^.

Adjusting tillage practices is an important strategy to reduce C and N losses from soil through mineralization and gaseous emissions^15,16^. Reduced tillage in terms of non-inversion tillage is also a suitable practice in organic agriculture if the soil conditions are favourable and weed pressure is low to maintaining soil structure and soil biota^17–20^. Reducing tillage can maintain or even increase the soil C stock^15,21^, but it can also be accompanied by yield losses in organic farming^20,22^, depending on soil conditions and the combination with other farming practices, e.g., the inclusion of leys in the crop rotation^22^, and cover crops to increase soil organic matter input^21,23^. Also, SOC gains through reduced tillage do not always translate to SOC stock increase when measuring deeper into the soil^20^.

Organic amendments, such as compost and mulch, add organic C and N to the soil, thereby improving soil quality for crop production and have the potential to increase the SOC stock^14,24–26^. This coupling of organic C and N inputs is specifically important in organic farming without mineral fertilizer application to maintain soil functioning^27^. But organic matter is also susceptible to accelerated biodegradation which is closely linked to decreasing C: N ratios^28^. The addition of external C and N sources to a field is critical due to the production of the required biomass, transfer to the field of interest, and potential C leakage effects elsewhere. Leakage effects can be avoided by focussing on zero net input organic systems, i.e., where the input of organic matter is limited to that amount of biomass C and N which could have been theoretically produced at the same site corresponding to the manure (N input) from one European livestock unit. This estimation can be used as a proxy for a closed system with on-site organic matter production^14^. The effects of organic amendments, reduced tillage, and other farming practices on subsoil SOC are interlinked and related to crop production^4,21^, which itself builds up organic C in the field. Net plant production (NPP) refers to the biomass of main crops as well as cover crops or catch crops integrated in crop rotations, which can provide a substantial in situ C input source^29^. While the main products are removed from the field, crop residues and cover crops, especially perennial plants such as grass‒legume mixtures, as well as roots and rhizodeposition, replenish SOC stocks^30^. Farming practices that increase biomass production are suitable for increasing net C sequestration but must be balanced with intensive soil management practices that deplete soil organic matter within the same year of crop production or along the crop rotation. This is specifically necessary in organic farming with lower yields and therefore reduced crop-derived C inputs. To impede a decline in SOC, an optimal organic scenario may therefore support widespread cover crops and enhanced residue recycling^31^.

This study was conducted in a field trial in Central Germany. Soil C inputs from NPP and C and N inputs from organic amendments of the different management practices were accumulated over ten years and related to a final SOC analysis in different soil layers and to the cumulative SOC stock with a depth of 1 m. The field was managed according to organic production principles, with a diversified crop rotation that includes cover crops. The main questions were as follows: (i) which farming practices or practice combinations increase the SOC stock? (ii) what is the contribution of different C sources to the SOC stock? (iii) do these management practices have the potential to increase subsoil SOC stocks? and (iv) can higher SOC stocks be achieved within the farms systems own productivity?

## Materials and methods

### Experimental field site

**Fig. 1:**
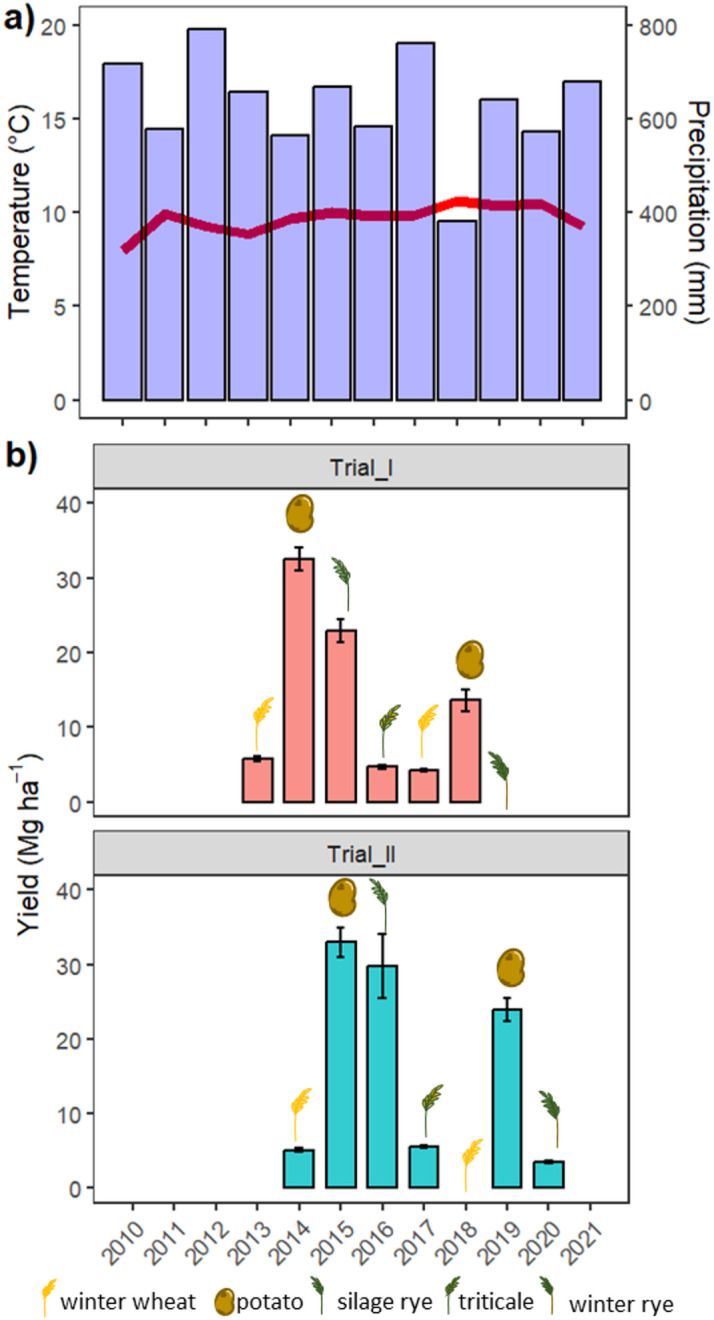
(**a**) Weather data and (**b**) yield dynamics (grain yield at 86% dry matter, silage rye as full plant yield and unsorted potato yield) over the experimental period in the two trials set up one year apart.

The ongoing experiment is located in Neu-Eichenberg in Central Germany at an experimental site at the University of Kassel (51°22‘N 9°54‘E). The annual precipitation at the site is 662 mm, with a mean annual temperature of 9.3 °C (30-year mean from 1990 to 2020 from a nearby weather station). Over the ten years of the experiment, the precipitation ranged from 560 to 790 mm, whereas 2018 was an untypically dry year, with only 380 mm (Fig. 1a). The soil is classified as Luvisol derived from loess with 13% clay, 84% silt, and 3% sand^32^ and a pH of 6.5. The site has been managed according to organic regulations (EU – Regulation EEC No. 2018/848) since 1988, which rely on organic fertilizers, the exclusion of synthetic plant protection agents and herbicides, and the pronounced inclusion of legumes and leguminous cover crops in the crop rotation.

The experimental setup consisted of two field trials established in 2010 (Trial_I) and 2011 (Trial_II), following the same crop rotation and management methods adapted to good agricultural practices, e.g., changes may have occurred according to weather conditions (Fig. 1a) and main crop performance (Fig. 1b) (Supplementary Tables S1 and S2; see also^18^). The two trials were set up one year apart to account for year-to-year weather variability.

**Fig. 2:**
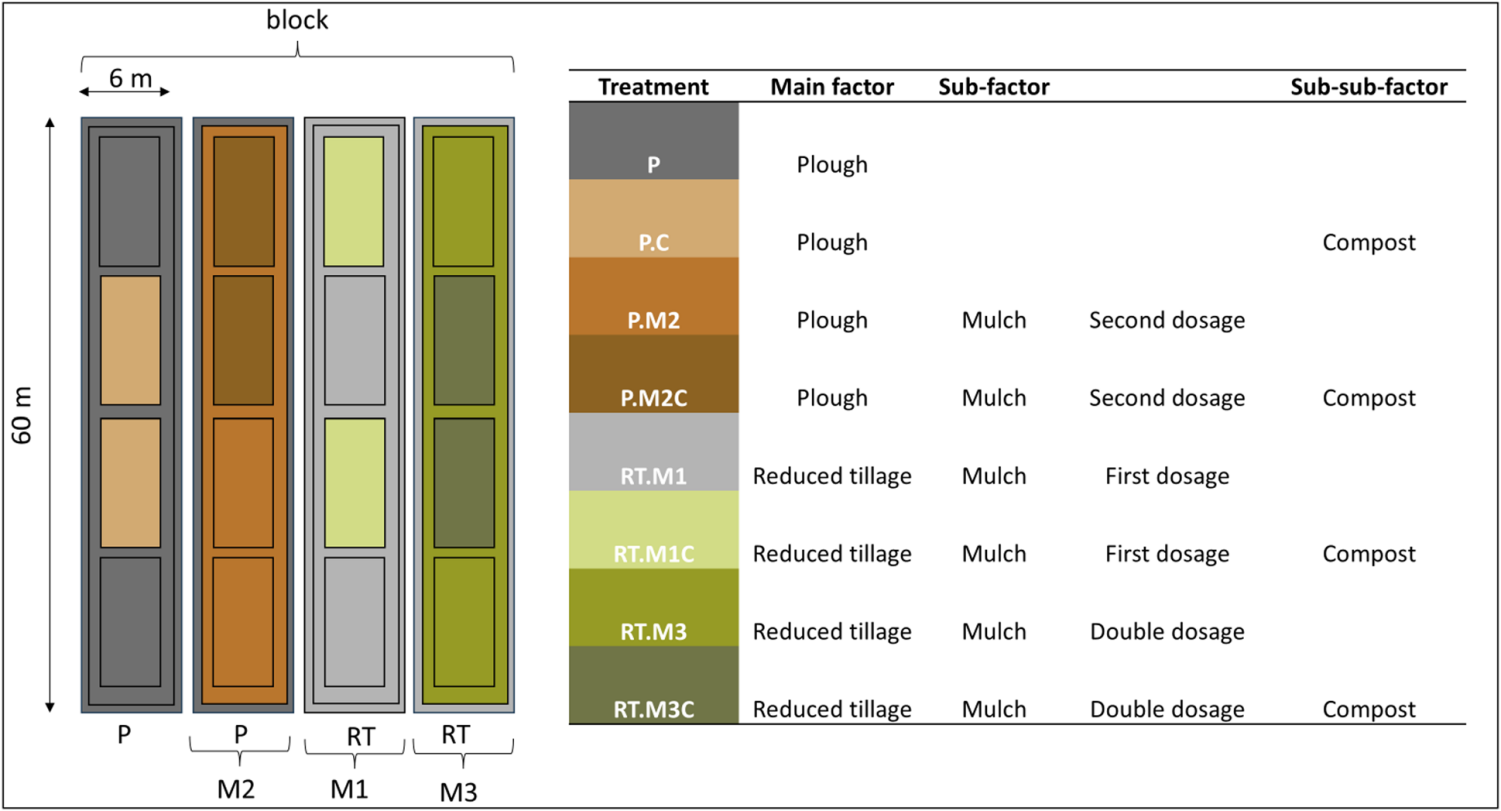
Scheme of one block and explanation of the experimental split-split-plot design with main factor plough (P) versus reduced tillage (RT), sub-factor mulch (M, where M1 and M2 refer to the first and second mulching, respectively, M3 to the double mulched treatments) and sub-sub-factor compost (C).

Each trial was divided into four blocks and three practices, i.e., reduced tillage, compost, and mulch application, following a split-split-plot design (Fig. 2). Each block contained two 6 × 60 m strips where inversion ploughing (P) down to a maximum depth of 25 cm was the main tillage practice and two strips with reduced tillage (RT) using a cultivator and/or rototiller (main factor tillage P vs. RT).

The organic mulch material (sub-factor mulch M) was applied only on the potato ridges, mainly as a crop protection measure^33^. In 2014 and 2015, respectively, rye-pea mulch (in Trial_I) and vetch triticale mulch (in Trial_II) was applied (first dosage) to the two strips per block with reduced tillage but not to the ploughed strips. In 2018 and 2019, respectively, vetch triticale mulch was applied (second dosage) again on two strips per block, one with reduced tillage and one with ploughing. Accordingly, plots received only the first mulch dosage (M1, i.e., RT.M1 and RT.M1C), only the second mulch dosage (M2, i.e., P.M2, P.M2C), or both dosages of mulch (M3, i.e., RT.M3, RT.M3C).

Finally, each strip was separated into four 15 m long plots. Yard waste compost from a commercial, quality-controlled composting facility was applied at mean dosages of 3 and 4 Mg ha^−1^ yr^−1^ in Trial_I and Trial_II, respectively, on two of the four plots (sub-sub-factor compost C). The carbon (C) and nitrogen (N) contents of the mulch were determined with a VarioMax CN Analyser (Elementar), and the compost was analysed via combustion and Kjeldahl-N, respectively (following the German compost quality standard RAL GZ 251)^34^. C and N content of organic amendments are shown cumulatively in Table 1 and in detail in Supplementary Table S2. To maintain nutrient balance, plots without compost application received equivalent amounts of potassium and phosphorus in the form of potassium sulphate and rock phosphate, respectively, in accordance with organic farming regulations^34^.

The farming practices were combined, resulting in a total of eight treatments ranging from ploughed plots with no regenerative practice (P) as a control to combinations of reduced tillage with double mulch and compost application (RT.M3C) (Table 1, Supplementary Table S2). Each treatment was repeated two times (*rep. plot*) per block (Fig. 2), resulting in 64 plots per trial and 128 plots in total.

To assess whether the external organic C and N inputs could have been theoretically produced as biomass at the same site, we used the animal stocking density as the European livestock unit (LU) ha^−1^ as a proxy of organic fertilizer N input to identify which treatment is a zero net input organic system. This assumes that a dairy cow (3,000 L milk without any additional concentrated feedstuff) produces 77 kg N year ^−1^ per year in the form of organic compounds^14^. A treatment with external inputs ≤ 1 LU ha ^−1^ is therefore identified as a zero net input organic system. This kind of system is theoretically able to produce the organic biomass on-site (according to its N input) that is used here as external organic C input. This assumption has to be taken with care and only as a proxy for organic N input and its related biomass C production. Values for zero net input organic systems may differ locally depending on pedoclimatic conditions.

**Table 1:** Compost and mulch applications according to dry matter (DM), and respective C and N inputs accumulated over ten years and summed for both mulch and compost (total). Treatments are plough (P), reduced tillage (RT), mulch (M, where M1 and M2 refer to the first and second mulching, respectively, M3 to the double mulched treatments) and compost (C) and their combinations. The C and N inputs in Trial_I and Trial_II differed because of the application of natural material with varying nutrient contents causing the deviation of the mean. The final column gives the hypothetical European livestock units (LU) needed per treatment to reach the amount of biomass N input.

Treatment	Compost			Mulch			Total C	Total N	LU
	Mg DM ha^−1^	Mg C ha^−1^	Mg N ha^−1^	Mg DM ha^−1^	Mg C ha^−1^	Mg N ha^−1^	Mg C ha^−1^	Mg N ha^−1^	
P	0	0	0	0	0	0	0	0	0
P.C	35	8.64 ± 0.41	0.50 ± 0.02	0	0	0	8.64 ± 0.41	0.50 ± 0.02	0.61
P.M2	0	0	0	17.90	7.52 ± 0.22	0.38 ± 0.02	7.52 ± 0.22	0.38 ± 0.02	0.49
P.M2C	35	8.64 ± 0.41	0.50 ± 0.02	17.90	7.52 ± 0.22	0.38 ± 0.02	15.84 ± 0.65	0.88 ± 0.00	1.10
RT.M1	0	0	0	19.37	6.27 ± 0.52	0.26 ± 0.03	6.27 ± 0.52	0.26 ± 0.03	0.34
RT.M1C	35	8.64 ± 0.41	0.50 ± 0.02	19.37	6.27 ± 0.52	0.26 ± 0.03	14.91 ± 0.93	0.76 ± 0.05	0.95
RT.M3	0	0	0	37.27	13.52 ± 0.31	0.65 ± 0.01	13.52 ± 0.31	0.65 ± 0.01	0.84
RT.M3C	35	8.64 ± 0.41	0.50 ± 0.02	37.27	13.52 ± 0.31	0.65 ± 0.01	22.16 ± 0.72	1.14 ± 0.03	1.44

### Crop rotation, crop management, yield estimation, and C allocation

In 2010 and 2011, respectively, the two trials started with two years of a grass-clover ley (cut and mulched), followed by a crop rotation of main crops and cover crops (Table 2, more in detail in Supplementary Table S2). Due to a severe drought in 2018, the crop rotations had to be adjusted through mulching and cover crop sowing. In September 2020 and 2021, respectively, rapeseed was sown in the two trials before the soil sampling.

**Table 2:** Crop rotation and main management activities over the course of the field trial. Years and main information refer to Trial_I. Any management adjustments in Trial_II, which started one year later, are indicated in the footnotes. Data availability shows which data of main crop yield and yield estimation of cover crops was available (√) or not available (-) and was estimated. A more detailed description is provided in Supplementary Table S2 and is given by Bilibio et al.^18^.

Year	Type	Crop	Main activity	Organic amendment	Data availability
2012	Cover crop	Grass clover	Mulching		-
2012			Fertilization	Compost	
**2013**	**Main crop**	**Winter wheat**	**Harvest; straw harvest and stubble incorporation**		**√**
2013	^1^Cover crop	Bristle oat, oilseed radish; winter vetch	Incorporation		√
2014			Fertilization	Compost	
2014			Mulch application	^2^Rye-pea mulch	
**2014**	**Main crop**	**Potato**	**Harvest**		**√**
**2015**	**Main crop**	**Silage rye**	**Harvest**		**√**
2015	Cover crop	Berseem clover	Mulching		-
**2016**	**Main crop**	**Triticale**	**Harvest; straw and stubble incorporation**		**√**
**2017**	^**3**^**Main crop**	**Winter wheat**	**Harvest; straw harvest and stubble incorporation**		^**3**^**√**
2017			Fertilization	Compost	
2018	Cover crop	Vetch triticale	Incorporation		√
2018			Fertilization	Compost	
2018			Mulch application	Vetch triticale mulch	
**2018**	**Main crop**	**Potato**	**Harvest**		**√**
2019	^4^Cover crop	Rye	Incorporation		-
2019	^5^Cover crop	Summer wheat	Mulching		√
2020	^6^Cover crop	Grass clover	Mulching		√
2020					-
2020			Fertilization	Compost	

^1^Cover crops were adjusted (various).

^2^Mulch material was changed: vetch-triticale mulch.

^3^Winter crop was mulched in spring and a cover crop was introduced; no main crop yield data available.

^4^Winter rye was kept as main crop.

^5^Winter rye was harvested as main crop: main crop yield data available.

^6^Cover crop was adjusted (vetch-triticale), second cover crop (various).

Main crops and related activities are in [bold].

The main crops, i.e., winter wheat (*Triticum aestivum*), potato (*Solanum tuberosum*), triticale (x *Triticosecale*), and green rye and winter rye (*Secale cereale*), were harvested and the yield was determined from a sampling area of 1.5 m by 10 m within each plot. Grain yield was recalculated at 86% dry matter, potato yield was measured directly from total unsorted potato yield and silage rye yield relates to full plant yield at harvest for silage production (Fig. 1b). Cover crop production was measured in a 1 m² subplot. Dry matter yield and crop C content were calculated using default crop-specific values^30^.

To assess the C input into the soil and the C that was exported from the field, we used equations established by Jacobs et al.^30^. and Skadell et al.^4^. to estimate the C in net plant production (NPP) and applied the provided C allocation factors (CA) for the different plant parts (see Jacobs et al.^30^, Supplementary Table S3). C removed from the field referred to the harvested yield export of the main crops and wheat straw, whereas stubble and other harvest residues (triticale straw) remained on the field and were incorporated. The additional C input into the soil was defined as the C from the roots and the rhizodeposition from the main crops. Cover crops were not harvested but were mulched and incorporated into the soil; therefore, total C from cover crops, including above- and belowground plant parts, was added as C input. If available, biomass data of cover crops were used for calculation (Table 2); otherwise, default values for C in NPP were used^30^ (Supplementary Table S3c). External C was added through mulch and compost (Table 1, Supplementary Table S2). Single-year C input data from crops, mulch and compost were collected for the entire measurement period (Trial_I: 2010–2020; Trial_II: 2011–2021) and cumulated.

### Soil sampling and analysis

The soil samples for chemical analysis were taken down to a depth of 1 m using a Nietfeld^®^ auger system mounted to a 3-point hitch of a tractor in October 2020 (Trial_I) and October 2021 (Trial_II) in the previously sown rapeseed. Each soil core (1 m length, 1.5 cm diameter) was separated into four depths: 0–10 cm, 10–30 cm, 30–50 cm, and 50–100 cm. To acquire sufficient soil material, five 1 m cores were taken per plot, and the topsoil down to a depth of 30 cm was also sampled two times with a manual auger (30 cm length, 4 cm diameter). The soil was bulked, resulting in one composite sample per plot and depth, which was crushed and sieved immediately (2 mm). A portion of ca. 300 g was dried at 40 °C for 48 h prior to analysis. SOC content was determined via temperature differentiation with a SoliTOC Analyser (Elementar) at 400°Cas were the total nitrogen (TN) content^35^.

Undisturbed soil samples were taken with cylinders (100 cm³) and special augers (Sample ring kit C, Eijkelkamp, NL) in the same year as the disturbed samples at one position per plot in randomly distributed plots in Trial_I (5 per block) and all plots in Trial_II at four depths for bulk density determination: 2.5–7.5 cm for 0–10 cm range; 17.5–22.5 cm for 10–30 cm; 37.5–42.5 cm for 30–50 cm; and 72.5–77.5 cm for 50–100 cm. The bulk density from the remaining plots per block in Trial_I was estimated from the mean per block and depth. In accordance with Krauss et al.^36^, the stock of soil organic carbon was calculated using soil bulk density data and expressed in Mg ha^−1^ for the respective soil layers

$$\begin{gathered} SOC\;stock\left[ {Mg\;ha^{{ - 1}} } \right] = SOC\;content\left[ {Mg\;100\;Mg^{{ - 1}} } \right] \hfill \\ \quad \quad \quad \quad \quad \quad \quad \quad \quad \quad \quad \quad *bulk\;density\left[ {Mg\;m^{{ - 3}} } \right]*soil\;layer\;thickness\left[ m \right]*100 \hfill \\ \end{gathered}$$

and the single layers were summed for the cumulative soil profile down to a depth of 1 m. We refrained from applying the equivalent soil mass approach because of lacking continuous soil bulk density along the depth profile. An interpolation as requested for the equivalent soil mass approach would increase uncertainty and pretend a precision, that is not provided by measured data. Instead, we decided to only report soil organic carbon stock using the fixed depth method. SOC changes over time, including sequestration, since onset of the trial could not be calculated because of missing initial SOC data down to a depth of 1 m. The pH of the soil samples was determined according to standard procedures with a 0.01 mol L^−1^ calcium chloride solution (Supplementary Table S4).

### Statistical analysis

We used linear mixed-effects models in R^37^ with the R package “lmerTest”^38^ to analyse the main effects of ‘*treatment’* (plough: P; plough and compost: P.C.; plough and mulch (M2: 2nd dosage): P.M2; plough, mulch (M2) and compost: P.M2C; reduced tillage and mulch (M1: 1st dosage): RT.M1; reduced tillage, mulch (M1) and compost: RT.M1C; reduced tillage and mulch (M3: double dosage): RT.M3; and reduced tillage, mulch (M3) and compost: RT.M3C) and ‘*trial’* (Trial_I and Trial_II) as fixed factors for the response variables (crop C_input_, crop C_export_, total C input (= crop C_input_ + mulch C + compost C), SOC content and SOC stock). No interaction of factors was included, as ‘*treatment’* effects were assumed to be consistent across the two parallel trials by design. To account for the block design, repeated plots per block, and the two trials, ‘*rep. plot nested into block nested into trial*’ was included as a random factor. For the soil-related response variables, the dataset was separated by depth and analysed individually, or the SOC stock was summed along the soil profile to a depth of 1 m.

Treatments with additional farming practices (RT, M, C and combinations) were tested against the control (P). The models were tested for homogeneity of variance and heteroscedasticity using standard diagnostic plots (residuals vs. fitted values, scale–location plots, and Q–Q plots, example in Supplementary Fig. S1). Visual inspection indicated that assumptions were met, and no data transformations were required. When significant differences were observed, a pairwise Tukey post hoc test was applied (R package “emmeans”^39^). To assess the relationships between variables, we calculated Pearson’s r.

The data in the tables and graphs (“ggplot2”^40^) are shown as the means and standard errors (SE) of treatments across plots and trials (“plyr”^41^) or as estimates and 95% confidence intervals (“modelsummary”^42^ and “tinytable”^43^) to show absolute differences from the control P.

## Results

### Cumulative C inputs and soil organic carbon after ten years

The C exported through harvested yield over the ten years was not significantly different between treatments, but the calculated C input arising from crop yield and C allocation factors differed slightly (Table 3, Supplementary Fig. S3). Conventional ploughing without external C input (P) generated C inputs from crops only (37.9 Mg C ha^−1^), which corresponded to 100% of the total C input in that treatment. In contrast, crop C input was highest in P.M2C (42.9 Mg C ha^−1^), i.e., + 13% crop-derived C compared to treatment P only, and total C input (58.8 Mg C ha^−1^) was 55% higher. The treatment with the highest total C input, RT.M3C (62.8 Mg C ha^−1^, 66% higher than P) had a crop C input of 40.6 Mg C ha^−1^ (+ 7% compared to P), while the remaining C input derived from the organic amendments.

A pairwise comparison of treatments revealed that compost application as a direct organic C source contributed to the total SOC stock compared with the corresponding control without compost (P vs. P.C: +8%; P.M2 vs. P.M2C: +11%; RT.M1 vs. RT.M1C: +4%; RT.M3 vs. RT.M3C: +7%), although the differences in the SOC stock between these treatments were not significant. Despite the high C load of the organic mulch, mulching did not increase the SOC stock (P vs. P.M2: -3%; RT.M1 vs. RT.M3: -3%; RT.M1C vs. RT.M3C: +0%) but had, in some cases, a slight adverse effect (non-significant). By reducing the tillage intensity, the SOC stocks significantly increased (P.M2 vs. RT.M1: +15%, *p* = 0.02), but non-significantly when also compost was applied (P.M2C vs. RT.M1C: +8). In this comparison of tillage practices, we assume that organic mulch applied during different years of crop rotation had no effect as shown before for the comparison of mulch treatments. Reducing tillage intensity without compost (RT.M1 and RT.M3) or ploughing with compost application (P.C) had similar effects on the SOC stock.

**Table 3:** Mean and standard error (SE) of crop C export and input, total C input (= crop C input + mulch C + compost C) accumulated over ten years, and soil organic carbon (SOC) stock down to 1 m depth separated for treatments (P: plough; C: compost; M: mulch, where M1 and M2 refer to the first and second mulching, respectively, M3 to the double mulched treatments; RT: reduced tillage). Letters indicate significant differences between treatments; significance levels of model effects are given below.

Treatment	Crop C export (sum over 10 years) [Mg C ha^−1^]			Crop C input (sum over 10 years) [Mg C ha^−1^]			Total C input (sum over 10 years) [Mg C ha^−1^]			SOC stock [Mg SOC ha^−1^]		
	Mean	SE		Mean	SE		Mean	SE		Mean	SE	
P	17.5	± 0.3	a	37.9	± 0.7	a	37.9	± 0.7	a	88.6	± 4.1	ab
P.C	17.7	± 0.5	a	38.5	± 0.9	a	47.1	± 1.1	b	95.8	± 3.7	abc
P.M2	17.3	± 0.5	a	40.3	± 0.9	ab	47.6	± 0.9	b	86.3	± 3.8	a
P.M2C	18.1	± 0.4	a	42.9	± 0.9	b	58.8	± 1.0	d	95.5	± 3.5	abc
RT.M1	16.3	± 0.5	a	37.5	± 1.6	ab	43.8	± 1.9	b	99.1	± 4.9	bc
RT.M1C	16.4	± 0.4	a	38.0	± 1.2	ab	53.0	± 1.9	c	102.7	± 3.8	c
RT.M3	17.2	± 0.5	a	40.5	± 1.3	ab	54.0	± 1.6	c	96.3	± 3.7	abc
RT.M3C	17.2	± 0.6	a	40.6	± 1.2	ab	62.8	± 1.9	d	103.2	± 4.2	c
Significance levels of model effects												
	0.097			< 0.001			< 0.001			< 0.001		

**Fig. 3:**
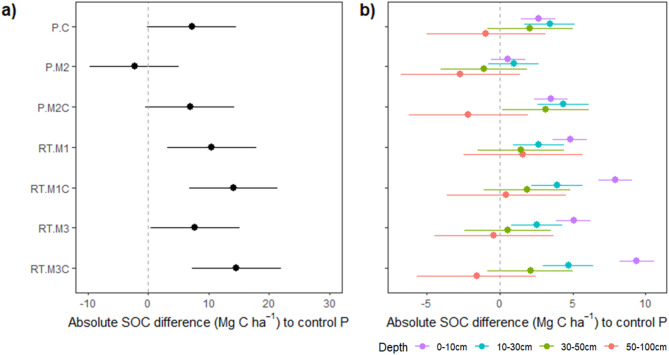
Difference (estimates and 95% confidence intervals) of (**a**) soil organic carbon (SOC) stocks as sum over the profile down to 1 m depth; (**b**) SOC stocks separated for soil layers (indicated by colours) of the treatments with one, two or three regenerative measures (C: compost; M: mulch, where M1 and M2 refer to the first and second mulching, respectively, M3 to the double mulched treatments; RT: reduced tillage, and their combinations) to the control (P: plough). The difference from the control is significant when the confidence intervals do not cross the dashed line.

Compared with the control treatment (P), the applied agronomic practices increased the cumulative SOC stocks to a depth of 1 m (Fig. 3a). The highest SOC stocks (+ 16% compared with those of the control) were obtained when compost and mulch application were combined with reduced tillage (RT.M1C and RT.M3C).

The effects of reduced tillage and compost were strongest in the topsoil: at the 0–10 cm and 10–30 cm soil depths, compost in ploughed plots (P.C) increased the SOC stocks by 19% and 12%, respectively, whereas reduced tillage (RT.M1) increased the SOC stock by 33% and 16%, respectively, and their combination (RT.M1C) increased the SOC stock by 57% and 14%, respectively, compared with those of the control P (Fig. 3b; Supplementary Table S4). Below 30 cm depth, no further differences were observed, implying that the topsoil effects dominated the effect along the 1 m profile. Compared with the control (P), organic mulch application in the ploughed treatment (P.M2) did not increase the SOC stock in any soil layer.

The SOC content followed the same pattern as the SOC stocks, whereas the effects were much greater in the 0–10 cm soil layer than in the other layers, resulting from a lower soil bulk density in the topsoil (Supplementary Fig. S3a; Supplementary Table S4). Additionally, the highest SOC contents were observed in the 0–10 cm layer in the RT treatments but not in the 10–30 cm soil layer.

The SOC and N contents in the soil samples were highly correlated (*r* = 0.96, *p* < 0.001) throughout the trials, soil layers, and treatments. The SOC-to-N ratio at the 10 cm depth was greater in P.C and P.M2C than in the control (P) but lower in RT.M3 (Supplementary Fig. S3b). The treatments P, P.C, P.M2, RT.M1, RT.M1C and RT.M3 were identified as zero net input organic systems with N inputs from compost and mulch ≤ 1 LU, whereas P.M2C and RT.M3C) were fed with additional input from outside the farm with 1.10 and 1.44 LU (Table 1).

## Relation of C input to SOC

**Fig. 4:**
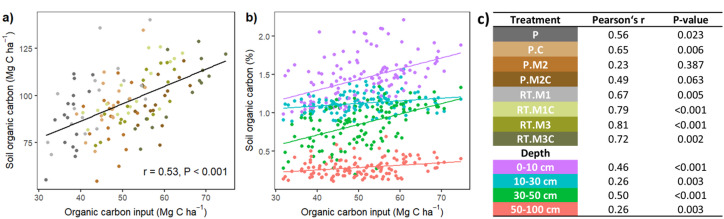
(**a**) Relation of total organic carbon input over ten years to soil organic carbon stocks as a sum over the profile down to 1 m depth, regression line across all treatment plots; (**b**) relation of total organic carbon inputs to the soil organic carbon content (%) in the four soil layers, regression lines for the soil layers across treatments; (**c**) legends and correlation coefficients (Peason’s r and P-value) for treatments (P: plough; RT: reduced tillage; C: compost; M: mulch, where M1 and M2 refer to the first and second mulching, respectively, and M3 refers to the double mulched treatments) and soil layer depths.

Carbon inputs from crops and organic amendments were drivers of the SOC stock (Fig. 4a). Except for P.M2, all the treatments presented positive correlations, with the highest correlation occurring at RT.M3, followed by RT.M1C and RT.M3C (Fig. 4c). The SOC content was highest in the uppermost soil layer (0–10 cm) and decreased with depth (Fig. 4b) and organic C input and SOC content were correlated in all layers (Fig. 4c).

## Discussion

The main organic C input in our experiment derived from the crops themselves, demonstrating that NPP was key to managing the SOC stock through crop residues, cover crops, roots, and rhizodeposition^30^. Reduced tillage combined with regular compost application (on average 0.7 Mg C ha^−1^ y^−1^), yielded the highest cumulative SOC stock down to a depth of 1 m after ten years confirming results by Krauss et al.^15^. In contrast, effects of mulch application were marginal despite its high organic C-loads (about 1.4 Mg C ha^−1^ y^−1^), indicating that high organic matter input does not necessarily translate directly into higher SOC stocks^24^.

### Farming practices affect cumulative SOC stocks

The cumulative SOC stocks in the plots that received compost were 4.3 to 9.0 Mg SOC ha^−1^ greater than those in the plots without compost. The C input from compost was in line with the application rate of 0.3 to 1.2 Mg C ha^−1^ y^−1^ of external C inputs described for organically and conventionally managed fields^14^.

The quality of external organic material plays a critical role in crop production and contributes to the SOC content^9,24^. The quality is related to the C: N ratio and other chemical properties (e.g., lignin or cellulose content)^9^ and in the case of compost depend on farming system specific storage and composting procedures^24^. These characteristics determine the turnover of the material, e.g., the decomposition rate, nutrient release and nutrient availability, which can be further related to soil biological quality and ultimately enhance crop production^24,44^. The organic amendments used in this study had an average C: N ratio of 18 (compost) and 22 (mulch). For mulch, this is a narrow ratio related to its high share of legumes compared to for example rye mulches with C: N ratio of 63^25^. The narrow C: N ratios implied fast N-mobilization rates and N-availability for plant production but also potential N-losses for example through gaseous emissions^25^. While the mulch material was known, the commercial yard waste compost was analysed for quality control and C: N ratio, but the various materials used and the decomposition process might have enhanced further effects through nutrient composition, e.g., on weed suppression^34^ and crop production.

The beneficial effect of high-quality organic amendments on SOC was demonstrated for farmyard manure and compost application in organic cropping systems in the topsoil^14,24^ and for farmyard manure down to 80 cm depth^4^. Like farmyard manure, compost has a direct fertilization effect and therefore enhances crop production^14^. In this study, crop-derived C was the biggest C input to the soil compared with the input from compost and mulch, although it must be pointed out that this data relies on calculation with C allocation factors and not directly measured values. The importance of crop- (especially root-) derived C input to soils and their contribution to SOC was shown in other studies^45,46^, but also the high uncertainty when using allometric functions for biomass C allocation^45^. Pedoclimatic variables but also crop variety and field management may affect root-to-shoot ratios^46^. Since varieties and pedoclimatic conditions remained similar over treatments in this study and overall yield was not different, we assumed that allocation factors were not strongly affected by treatments, but real values might have different from applied values potentially resulting in an underestimation of crop-derived C, since root carbon allocation was found to be higher under organic than under conventional management^46^, where the used values were coming from^30^.

While SOC is an output of plant production, maintaining and increasing SOC may also increase yields, at least to a certain extent, due to the strong correlation of SOC with soil N and other soil quality characteristics related to SOC^21^, e.g., water retention^3^. Releasing N from soil organic matter by mineralization drives nutrient recycling processes in the soil^24^ and decreases the need for external N input^47^. In the same process, also SOC is mineralized through soil microbial activity indicated by basal respiration rates. However, microbial carbon turnover of soil organic matter might be precursor of long-term stabilization of non-respired carbon in mineral-bound carbon pools^24^.

Organic mulch can increase crop growth directly through mineralization and nutrient release or indirectly through soil microclimatic effects, e.g., temperature buffering and residual water^17,25^, and plant protection in organic production systems, which was specifically shown for potato^33,48^. Both the incorporation of mulch material into the soil directly and via enhanced plant production may contribute to SOC. In our study, mulching did not result in enhanced plant production and C input by crops over the ten-year period nor higher SOC stocks. That the organic C application of the mulch was not adding C to the SOC stock implies a loss of C. Again, characteristics of the mulch, like the C: N ratio, play a role in underlying soil processes, such as the release or fixation of available N and soil microbial activity^17,28^, and probably increase N and C losses through gaseous emissions^25^. The narrow C: N ratio (17 to 27) of the leguminous crop mixture used as mulch in this study may have provoked a fast turnover^28,49^.

Reduced tillage had no positive effect on crop production in this trial but did also not decrease overall productivity, as observed in other organic field trials^22^. Greater SOC stocks resulting from reduced tillage compared with those resulting from ploughing can be explained through enrichment by plant production and crop-derived C input, as well as by reduced disturbance to the soil, maintaining aggregates and soil quality^15,50^, soil hydraulic properties^3^ and probably reducing microbial C mineralization, followed by lower CO_2_ emissions^36,51^.

Combining reduced tillage with compost application yielded the highest SOC stocks (RT.M1C and RT.M3C), as confirmed by Krauss et al.^50^, combining the benefits of reduced soil disturbance with high C inputs from compost itself and through crop fertilization.

### Farming practices had only minor effects on subsoil SOC

Skadell et al.^4^ reported from ten long-term experiments that management practices affect not only topsoil SOC but also SOC below a depth of 30 cm, with most of these effects occurring mainly in the 30–50 cm soil layer. The most effective practices among the different experiments included fertilization and organic input practices, implying that the effect was related to C input through crop production via fertilization and the incorporation of organic matter, here farmyard manure and straw^4^. Other strategies include diversified crop rotations and perennial or semi-perennial plants such as alfalfa^52^. Beside deep-rooting plants and their exudates, also bioturbation and leaching of dissolved organic C can be potential mechanism for this subsoil organic C increase^53^. In our trial, we did not observe significant effects in the treatments on SOC stocks and content below a depth of 30 cm compared with the control, the topsoil effects on the SOC stock were strong enough to dominate the effect over the whole soil profile; however, an increase in SOC content below a 30 cm depth with increasing C input (Fig. 3b) implied some effects on plant production in the subsoil. In contrast to other observations that a gain in topsoil SOC is accompanied with lower SOC in the deeper soil layers^20^, we did not find a decrease in SOC below 30 cm.

The highest SOC stocks were obtained in the topsoil layers when reduced tillage was accompanied by compost and double mulch application (RT.M3C). Here, most of the effects on SOC stocks occurred in the surface soil at depths of 0–10 cm, where organic matter was directly incorporated and where most plant roots are located but where the greatest disturbance to the soil was caused by the reduced tillage. This explains the strong stratification of the 0–10 cm layer with highest SOC stocks compared to soil layers below with reduced tillage, as by Krauss et al.^50^. In contrast, ploughing transferred the C inputs of the compost, organic mulch, crop residues, and cover crops to depths of 10–30 cm, where higher SOC stocks and content was observed compared to the 0–10 cm soil layer (Fig. 3b).

Based on our experiments, compost application and reduced tillage, especially when combined, appear to be promising practices for increasing the total SOC stock. However, short-term management changes, e.g., the intensification of crop-row management, can rapidly affect SOC stocks in the topsoil and revert previous improvements in SOC^6^. Subsoils below the common management depth of 20–30 cm are less exposed to disturbance and typically have higher clay content and bulk density and can therefore promote long-term C stabilization and storage^8,52^ and should therefore be considered for carbon farming approaches. We observed an increase in SOC in subsoils with increasing organic C input probably related to at least some deep-rooting crop species or varieties that deposit C in deep layers through slow root decomposition and root exudates^54^, to deep dwelling anecic earthworms translocating organic matter to greater depth^55^, or leaching of dissolved organic carbon^53^. Other potential strategies to increase SOC sequestration in subsoils exist, including infrequent deep ploughing below 30 cm depth^56^ or biochar burial^57^ but were not applied in this study.

Previous studies conducted at the same experimental trials confirmed the positive impact of the combination of reduced tillage and compost application on soil quality parameters in the main crop rooting zone, e.g., on soil physical parameters^3,18^, soil microbial activity and abundance^58^, and soil suppressiveness^59^.

In 2018, severe drought reduced crop yields in Europe. Legacy effects and another year of below average precipitation impact soil moisture and groundwater recharge^60^. In our trials, the 2018 drought drastically reduced potato yield, and wheat production failed and limited winter rye and potato production in 2019, leading to reduced NPP and crop-derived C inputs compared with those in wetter years such as 2014 and 2015. Despite lower C input during drought, also soil microbial respiration can be limited, reducing C loss through emissions. The interactions of soil moisture and SOC dynamics are complex and context-specific depending also on farming practices^61^. Mulching can conserve water in the soil and at least to some extent support crop production under dry conditions^25^ and maintain microbial activity^17^. For instance, we observed higher potato yields with mulch in the drought years 2018 and 2019 in our trials (data not shown).

Decreases in C inputs through reductions in main crop production under climate change-induced droughts and heat during the main cropping season^62^ may be partially balanced by organic amendments to sustain SOC levels and soil quality^31,63^. The gross C input from potato is generally low and intensive ridge cultivation for potato production is known to further reduce SOC^64,65^ due to soil disturbance and mineralization. Compost application added external C aiming to balance C losses somehow^27^.

Deep-rooting mixtures of cover crops can have additional beneficial effects on deep SOC translocation and storage, and their effects on soil quality have the potential to stabilize yields under changing climatic conditions^66^. Cover crops during wintertime have the potential to use off-season precipitation to increase NPP and in situ C inputs. However, the water demands of cover crops over the winter period may interfere with the needs of succeeding main crops in dry years, requiring their timely termination, e.g., by using frost-sensitive cover crops, to reduce water competition^67^. Early termination of cover crop growth in turn reduces biomass production and SOC accumulation^68^. Since cover crops were an integral component of the crop rotation, their specific effects on SOC accumulation and deep C storage could not be evaluated individually.

### SOC accumulation within the system boundaries

The treatments with the highest SOC stock compared with the control were not identified as zero net input organic systems (Table 1) but relied on organic matter that was produced elsewhere. Similar results were found by Krause et al.^24^. where only an organic input of 1.4 LU was contributing to SOC sequestration in a long-term experiment. We identified other treatments as zero net input organic systems that still obtained higher SOC stocks than the control, but their C sequestration potential or long-term storage has to be confirmed in a follow-up study. Since net plant production was identified as the main input source for organic carbon to the soil, farming practices that enhance crop production still following organic principles must be supported, including deep-rooting crops and cover crop mixtures, while reducing negative impacts, for instance during dry spells. Reduced tillage, if it is practical under prevailing soil conditions^22^, could be a suitable option to increase SOC stocks without additional input as it is less harmful to anecic earthworm populations to translocate organic matter to depth^55^. When other practices are identified to increase SOC, it might be possible to reduce the application of organic amendments like compost and mulch to an amount ≤ 1 LU, while still supporting their beneficial effects on soil and plant protection, and fertilization in organic farming. Although the assumption of zero net organic input systems has to be interpreted with care and only as a proxy, they give an idea of the on-farm production within the farm’s boundaries and circularity.

Practice-based carbon rewarding and MRV (Monitoring, Reporting and Verification) schemes should consider the origin and nutrient content of organic amendments and the effects of the farming practices on deeper soil layers to account for leakage and long-term C storage.

Unlike many conventional farming experiments where farming practices are studied in isolation^4^, the design of this trial integrates diverse crop rotation and crop-specific management strategies that align it with organic farming practices and is consistent with other long-term organic field experiments, such as the DOK-trial^24,44^, the-Frick trial^50^ in Switzerland, and the OAFEG-trial in Germany^22,69^. Long-term trials, typically defined as being older than 20 years, are supposed to provide more accurate information^70^, especially on long-term effects such as C sequestration^64^. The SOC measurements after ten years in the present field trial refer to a medium-term experiment but they provide valuable information on the applied practices, especially in the topsoil, and trends in the subsoil after changing practices. In long-term trials, SOC and C sequestration after more than 20 years^44^ can be revised when monitoring continues^24^, implying the need for even longer experiments in organic and regenerative agriculture with continuous monitoring and revision of practices, data, and interpretation.

## Conclusions

Reduced tillage and compost application increased the SOC stocks, especially when combined, whereas mulch had no effect. Crop and cover crop production in situ contributed the most to the SOC stock through the accumulation of residues, roots and root exudates over the ten years of observation Compost as an organic amendment that increases crop production has a good potential to contribute to the SOC stock, but application must be balanced to reduce the input of material produced outside the farm boundaries to avoid leakage effects. Reduced tillage instead is less critical in terms of system boundaries but can be accompanied with other drawbacks on productivity.

While topsoil SOC was responsive to management, no significant changes in farming practices compared with those in the control (ploughing without organic amendments) were observed in the subsoil, although we found that cumulative SOC stocks and SOC content also in subsoil layers increased with increasing input of organic C.

Regenerative organic agriculture relying on the here studied farming practices has a positive effect on soil quality including N and SOC stocks in the topsoil but may not contribute to long-term carbon mitigation in its current form. To increase long-term carbon storage in deep soil layers, future organic management should emphasize the use of deep-rooting main crop cultivars and cover crop mixtures while considering trade-offs, e.g., water demand.

Carbon sequestration and losses through CO₂ emissions have not yet been measured, limiting the calculation of the total carbon budget. Follow-up investigations in the same trial will assess both carbon sequestration after five years and gaseous emissions related to farming practices to estimate full carbon balance.

## Supplementary Information

Below is the link to the electronic supplementary material.

Supplementary Material 1

## Data Availability

The datasets generated and/or analysed during the current study will be available at BONARES repository.
