# Supplementary material for: Soil organic carbon stocks after ten years of reduced tillage, compost and mulch application in temperate organic agriculture
Source: Sci Rep. 2026 Mar 5;16:8260. doi: 10.1038/s41598-026-42050-9 (PMC12963617; doi:10.1038/s41598-026-42050-9)
Supplement: Supplementary file 1 — Supplementary Material 1 [file 41598_2026_42050_MOESM1_ESM.pdf]

Supplementary Material

Soil organic carbon stocks after ten years of reduced tillage, compost and mulch application in temperate organic agriculture

[1]Wiebke Niether*

[2]Simeon Leisch-Waskönig

[2]Maria R. Finckh

[2]Stephan Martin Junge

[3]Carolina Bilibio

[4]Stephan Peth

[5]Jan Henrik Schmidt

[1,6]Juliet Wanjiku Kamau

[1,7]Andreas Gattinger

[7]Institute of Organic Agriculture (FiBL), Kasseler Strasse 1a, 60486 Frankfurt, Germany

[1]Organic Farming with Focus on Sustainable Soil Use, Justus-Liebig-University of Giessen, Karl-Gloeckner-Str. 21 C, 35394 Giessen, Germany; wiebke.niether@agrar.uni-giessen.de;

[2]Ecological Plant Protection, University of Kassel, Nordbahnhofstrasse 1a, 37213 Witzenhausen, Germany

[3]Soil Science, University of Kassel, Nordbahnhofstrasse 1a, 37213 Witzenhausen, Germany;

[4]Institute of Earth System Sciences, Leibniz-University-Hannover, Herrenhäuser Strasse 2, 30419 Hannover, Germany

[5]Institute for Epidemiology and Pathogen Diagnostics, Julius Kühn Institute (JKI) - Federal Research Centre for Cultivated Plants, Messeweg 11/12, 38104 Braunschweig, Germany

[6]Center for Development Research (ZEF), University of Bonn, Genscherallee 3, 53113 Bonn, Germany

Table S1 Yield dynamics (grain yield at 86% dry matter, silage rye as full plant yield and unsorted potato yield, cover crops as total dry matter) over the experimental period in the two trials set up one year apart (mean and standard error).

		Trial_I yield			Trial_II yield	
**Year**	**Crop**	**Mg ha^{-1} y^{-1}**		**Crop**	**Mg ha^{-1} y^{-1}**	
2010	Cover crop	NA	NA			
2011	Cover crop	NA	NA	Cover crop	NA	NA
2012	Cover crop	NA	NA	Cove rcrop	NA	NA
2013	Winter wheat	5.7	± 0.4	Cover crop	NA	NA
	Cover crop	0.5	± 0.1			
2014	Potato	32.5	± 1.5	Winter wheat	5.1	± 0.2
				Cover crop	0.4	± 0.2
2015	Silage rye	22.9	± 1.5	Potato	33.0	± 1.9
2016	Triticale	4.6	± 0.2	Silage rye	29.7	± 4.3
2017	Winter wheat	4.3	± 0.2	Triticale	5.6	± 0.2
2018	Potatoe	13.6	± 1.5			
	Cover crop	2.0	0.1	Cover crop	NA	NA
2019	Cover crop	6.0	0.9	Potatoe	23.9	1.6
				Cover crop	5.3	0.4
2020	Cover crop	3.8	0.2	Winter rye	3.5	0.2
2021				Cover crop	4.1	0.8

Table S2 Crop rotation and main activities, i.e., incorporation of organic material, compost and mulch application, in the two field trials Trial_I and Trial_II over the evaluation period. A detailed description on the field operations from 2010 is given by (Bilibio et al. 2023).

Trial_I				Compost				Mulch				
Date	Type	Crop	Main activity	Mg DM ha^{-1}	Mg C ha^{-1}	Mg N ha^{-1}	C:N-ratio	Mg DM ha^{-1}	Mg C ha^{-1}	Mg N ha^{-1}	C:N-ratio	mulch type
2012-09	Cover crop	Grass clover	Mulching									
2012-10			Fertilization	5	1.45	0.09	16					
2012-10	Main crop	Winter wheat	Undersowing									
2013-08	Main crop	Winter wheat	Harvest									
2013-08			Straw and stubble incorporation									
2013-11	Cover crop	Bristle oat, oilseed radish; winter vetch	Incorporation									
2014-04			Fertilization	10	2.08	0.13	16					
2014-05	Main crop	Potato	Mulch application					12.23	4.25	0.16	27	Rye-pea
2014-09	Main crop	Potato	Harvest									
2015-05	Main crop	Silage rye	Harvest									
2015-10	Cover crop	Berseem clover	Mulching									
2016-07	Main crop	Triticale	Harvest									
2016-07			Straw and stubble incorporation									
2017-05	Main crop	Winter wheat	Undersowing									
2017-08	Main crop	Winter wheat	Harvest									
2017-08			Straw and stubble incorporation									

2017-09			Fertilization	5	1.44	0.76	19					
2018-04	Cover crop	Vetch triticale	Incorporation									
2018-04			Fertilization	5	1.32	0.72	18					
2018-05	Main crop	Potato	Mulch application					20.90	8.09	0.46	18	Vetch triticale
2018-09	Main crop	Potato	Harvest									
2019-04	Cover crop	Rye	Incorporation									
2019-07	Cover crop	Summer wheat	Mulching									
2020-07	Cover crop	Grass clover	Mulching									
2020-08			Fertilization	5	0.77	0.55	14					
2020-08			Undersowing									
			**SUM**	**30**	**7.05**	**0.42**		**33.13**	**12.34**	**0.61**		

**Trial_II**				**Compost**				**Mulch**				
**Year**	**Type**	**Crop**	**Main activity**	**Mg DM ha^{-1}**	**Mg C ha^{-1}**	**Mg N ha^{-1}**	**C:N-ratio**	**Mg DM ha^{-1}**	**Mg C ha^{-1}**	**Mg N ha^{-1}**	**C:N-ratio**	**mulch type**
2013-09	Cover crop	Grass clover	Incorporation									
2013-10			Fertilization	5	1.85	0.08	25					
2013-10	main crop	Winter wheat	Undersowing									
2013-08	main crop	Winter wheat	Harvest									
2013-08			Straw and stubble incorporation									
2014-11	Cover crop	Various	Incorporation									
2015-04			Fertilization	5	0.85	0.07	13					
2015-05	main crop	Potato	Mulch application					26.51	8.29	0.30	22	Vetch triticale
2015-09	main crop	Potato	Harvest									
2016-05	main crop	Silage rye	Harvest									
2016-08	Cover crop	Berseem clover	Mulching									

2017-08	main crop	Triticale	Harvest									
2017-08			Straw and stubble incorporation									
2018-06	Cover crop	Winter wheat	Mulching									
2018-09	Cover crop	Various	Mulching									
2018-10			Fertilization	5	1.38	0.06	23					
2019-05	Cover crop	Vetch triticale	Mulching									
2019-05			Fertilization	10	2.40	0.10	24					
2019-06	main crop	Potato	Mulch application					14.90	0.641	0.30	21	Vetch triticale
2019-09	main crop	Potato	Harvest									
2020-07	main crop	Winter rye	Harvest									
2020-07			Straw and stubble incorporation									
2021-06	Cover crop	Vetch triticale	Mulching									
2021-08	Cover crop	Various	Mulching									
2021-08			Fertilization	10	2.94	0.21	14					
2021-08			Undersowing									
			**SUM**	**35**	**9.42**	**0.51**		**41.41**	**1.47**	**0.68**		

Table S3 a) Estimation of organic carbon (C_{org}) exports and inputs from main crops (MP: fresh matter yield of main product), cover crops (CC) and organic amendment mulch and compost; b) main crop-specific C_{org} allocation factors according to Jacobs et al. (2020) -Table 1; c) default annual above ground- and belowground C_{org} net primary production (NPP) from cover crops according to Jacobs et al. (2020) -Table S1. DM: dry matter content; C_{MP}, $C_{compost}$, C_{mulch}: carbon content of the respective organic material; HR: harvest residue; ST: stubble; R: roots; RD: rhizodeposition. Harvest residues (except wheat straw), stubbles and cover crops remained on the field.

a)

Explanation	Equation	Reference
C_{org} leaving the field	$Total.C_{export} = Product_{MP} * DM_{MP} * C_{MP}$	(Jacobs *et al.* 2020) Equations (9)-(12)
C_{org} in main crops aboveground aboveground	$MP.NPP_{above} = (Product_{MP} + HR_{MP}) * DM_{MP} * C_{MP} + (Product_{MP} + HR_{MP}) * DM_{MP} * \frac{C_{MP}}{CA_{MP}} * CA_{HR} + (Product_{MP} + HR_{MP}) * DM_{MP} * \frac{C_{MP}}{CA_{MP}} * CA_{ST}$	(Jacobs *et al.* 2020) Equation (6)
C_{org} in main crops belowground	$MP.NPP_{below} = (Product_{MP} + HR_{MP}) * DM_{MP} * \frac{C_{MP}}{CA_{MP}} * CA_{R} + (Product_{MP} + HR_{MP}) * DM_{MP} * \frac{C_{MP}}{CA_{MP}} * CA_{RD}$	(Jacobs *et al.* 2020) Equation (7)
C_{org} in main crops total	$MP.NPP_{total} = MP.NPP_{below} + MP.NPP_{above}$	(Jacobs *et al.* 2020)
C_{org} input from main crops aboveground	$MP.C_{input-above} = MP.NPP_{total} * CA_{HR} + MP.NPP_{total} * CA_{ST}$	(Skadell *et al.* 2023) Equation (A.4)
C_{org} input from main crops belowground	$MP.C_{input-below} = MP.NPP_{total} * CA_{R} + MP.NPP_{total} * CA_{HR}$	(Skadell *et al.* 2023) Equation (A.5)
C_{org} input from cover crops aboveground	$CC.C_{input-above} = CC.NPP_{above}$	(Jacobs *et al.* 2020) Table S1
C_{org} input from cover crops belowground	$CC.C_{input-below} = CC.NPP_{below}$	(Jacobs *et al.* 2020) Table S1
C_{org} in compost	$Compost.C_{input} = Compost * DM_{compost} * C_{compost}$	Compost analyses
C_{org} in mulch material	$Mulch.C_{input} = Mulch * DM_{mulch} * C_{mulch}$	Mulch analyses
Total input of C_{org} to the soil	$Total.C_{input} = MP.C_{input-above} + MP.C_{input-below} + CC.C_{input-aboce} + CC.C_{input-below} + Compost.C_{input} + Mulch.C_{input}$	

b)

Main crop		DM_{MP}	C_{MP}	C_{HR}	CA_{MP}	CA_{HR}	CA_{ST}	CA_{R}	CA_{RD}
Winter wheat	*Triticum aestivum*	0.86	0.46	0.46	0.417	0.284	0.050	0.190	0.059
Potato	*Solanum tuberosum*	0.22	0.47	0.47	0.798	0.160	0	0.033	0.010
Grain rye	*Secale cereale*	0.86	0.47	0.47	0.404	0.308	0.054	0.178	0.055
Triticale	x *Triticosecale*	0.86	0.45	0.46	0.421	0.326	0.058	0.149	0.046

Default values from[31]-Table 1

c)

Cover crop		$CC.NPP_{above}$	$CC.NPP_{below}$
Grass clover	*Trifolium* spp., *Lolium* sp.	1.6	0.7
Bristle oat, oilseed radish; winter vetch (mixture of legumes and nonlegumes)	*Avena strigosa*, *Raphanus sativus* var., *Vicia villosa*	2.4	0.8
Berseem clover	*Trifolium alexandrinum*	1.2	0.4
Vetch triticale	*Vicia villosa*; x *Triticosecale*	2.4	0.8
Rye cover crop	*Secale cereale*	2.6	0.7
Wheat cover crop	*Triticum aestivum*	2.6	0.7
Grass clover mixture	Various species	1.6	0.7
Various	Various species	2.4	0.8

Default values from Jacobs *et al*. 2020 - Table S1

Table S4 Mean and standard error (SE) of soil organic carbon (SOC) stocks and concentrations in two trials across treatments and of the treatments (P: plough; C: compost; M: mulch, where M1 and M2 refer to the first and second mulching, respectively, M3 to the double mulched treatments; RT: reduced tillage) across trials separated for soil layers. Letters indicate differences between trials and treatments, respectively.

Treatment	Depth [cm]	SOC [Mg C ha-1] mean	SE		SOC [%] mean	SE	Bulk density [g cm^{-3}] mean	SE	pH mean	SE
P	0-10	13.8	±0.2	a	1.09	±0.02	1.27	±0.02	6.40	±0.05
P.C	0-10	16.5	±0.6	b	1.33	±0.04	1.23	±0.02	6.48	±0.05
P.M2	0-10	14.4	±0.3	a	1.12	±0.02	1.28	±0.02	6.37	±0.06
P.M2C	0-10	17.3	±0.5	bc	1.40	±0.03	1.24	±0.02	6.55	±0.06
RT.M1	0-10	18.6	±0.3	c	1.47	±0.02	1.27	±0.01	6.53	±0.09
RT.M1C	0-10	21.7	±0.8	d	1.75	±0.05	1.24	±0.02	6.62	±0.06
RT.M3	0-10	18.9	±0.5	c	1.52	±0.02	1.24	±0.02	6.54	±0.07
RT.M3C	0-10	23.2	±0.8	d	1.87	±0.05	1.24	±0.02	6.64	±0.06
P	10-30	28.1	±0.6	a	1.03	±0.02	1.37	±0.01	6.41	±0.07
P.C	10-30	31.5	±0.6	bc	1.16	±0.02	1.35	±0.01	6.49	±0.05
P.M2	10-30	29.0	±0.7	ab	1.06	±0.02	1.37	±0.01	6.40	±0.06
P.M2C	10-30	32.5	±0.9	c	1.21	±0.03	1.35	±0.01	6.52	±0.05
RT.M1	10-30	30.7	±0.5	abc	1.10	±0.02	1.40	±0.02	6.38	±0.06
RT.M1C	10-30	32.0	±0.7	c	1.16	±0.03	1.38	±0.02	6.47	±0.04
RT.M3	10-30	30.6	±0.8	abc	1.09	±0.03	1.40	±0.02	6.47	±0.04
RT.M3C	10-30	32.7	±1.0	c	1.19	±0.04	1.38	±0.01	6.46	±0.05
P	30-50	23.9	±1.8	a	0.81	±0.06	1.47	±0.01	6.35	±0.07
P.C	30-50	25.9	±1.9	a	0.87	±0.06	1.48	±0.01	6.49	±0.06
P.M2	30-50	22.8	±2.0	a	0.77	±0.07	1.47	±0.01	6.36	±0.05
P.M2C	30-50	27.0	±1.6	a	0.93	±0.05	1.45	±0.02	6.48	±0.04
RT.M1	30-50	25.3	±1.9	a	0.87	±0.06	1.45	±0.01	6.40	±0.05
RT.M1C	30-50	25.7	±2.2	a	0.88	±0.07	1.46	±0.01	6.44	±0.03
RT.M3	30-50	24.4	±1.9	a	0.84	±0.06	1.45	±0.01	6.35	±0.08
RT.M3C	30-50	25.9	±2.2	a	0.89	±0.08	1.46	±0.02	6.42	±0.05
P	50-100	22.9	±2.3	a	0.29	±0.03	1.57	±0.03	6.72	±0.09
P.C	50-100	21.9	±2.3	a	0.29	±0.03	1.52	±0.02	6.81	±0.08
P.M2	50-100	20.2	±2.0	a	0.26	±0.03	1.52	±0.01	6.81	±0.09
P.M2C	50-100	20.7	±1.7	a	0.27	±0.02	1.53	±0.02	6.68	±0.08
RT.M1	50-100	24.5	±3.2	a	0.32	±0.04	1.53	±0.02	6.70	±0.10
RT.M1C	50-100	23.3	±2.3	a	0.30	±0.03	1.52	±0.01	6.67	±0.09
RT.M3	50-100	22.5	±2.1	a	0.29	±0.03	1.53	±0.01	6.76	±0.08
RT.M3C	50-100	21.3	±2.3	a	0.27	±0.03	1.54	±0.02	6.71	±0.10

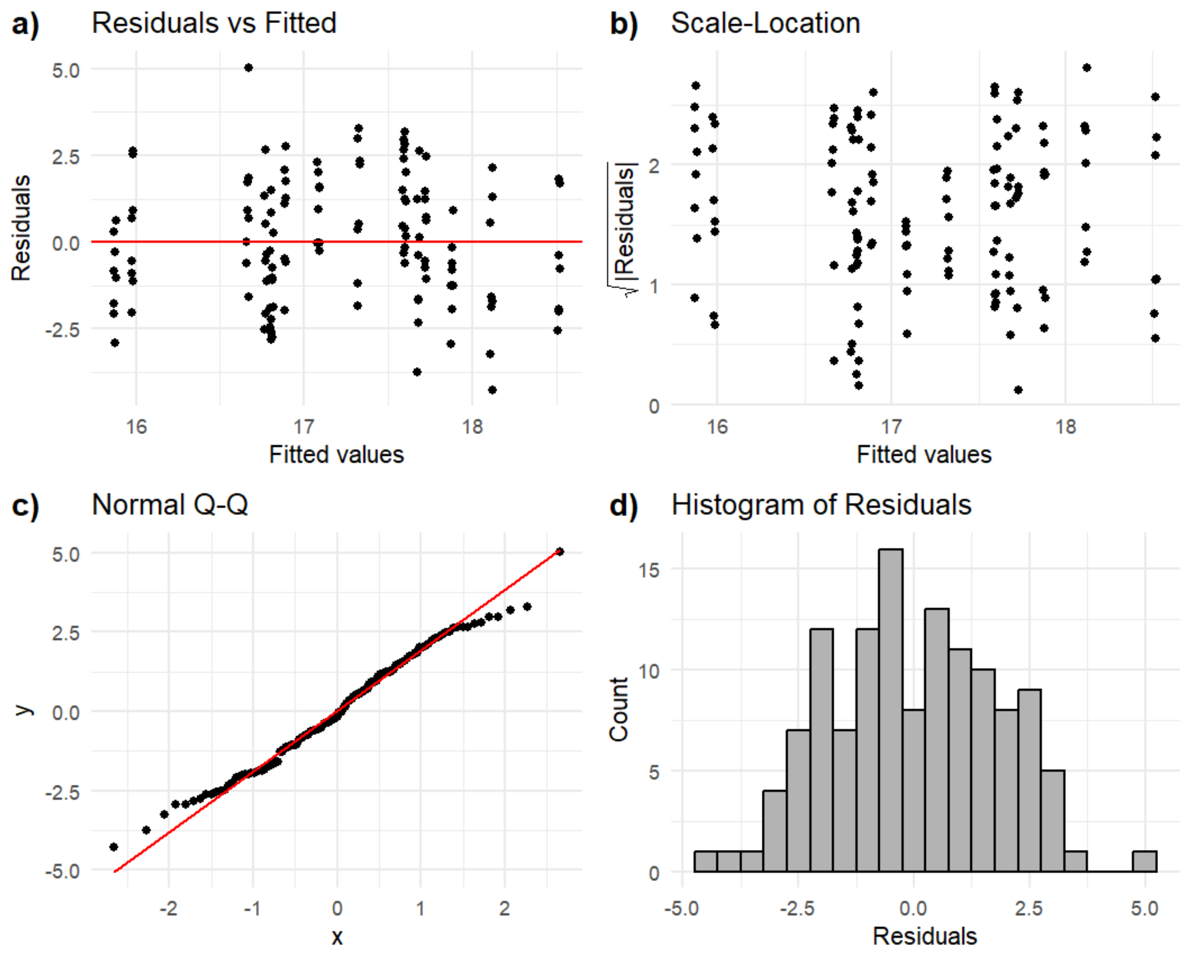

Figure S1 Diagnostic plots for testing for normality, homogeneity of variance, and heteroscedasticity of the model for C export analysis:

mod<-lmer((C_export)~ treatment + trial +(1|trial/block/rep.plot) , data=data).

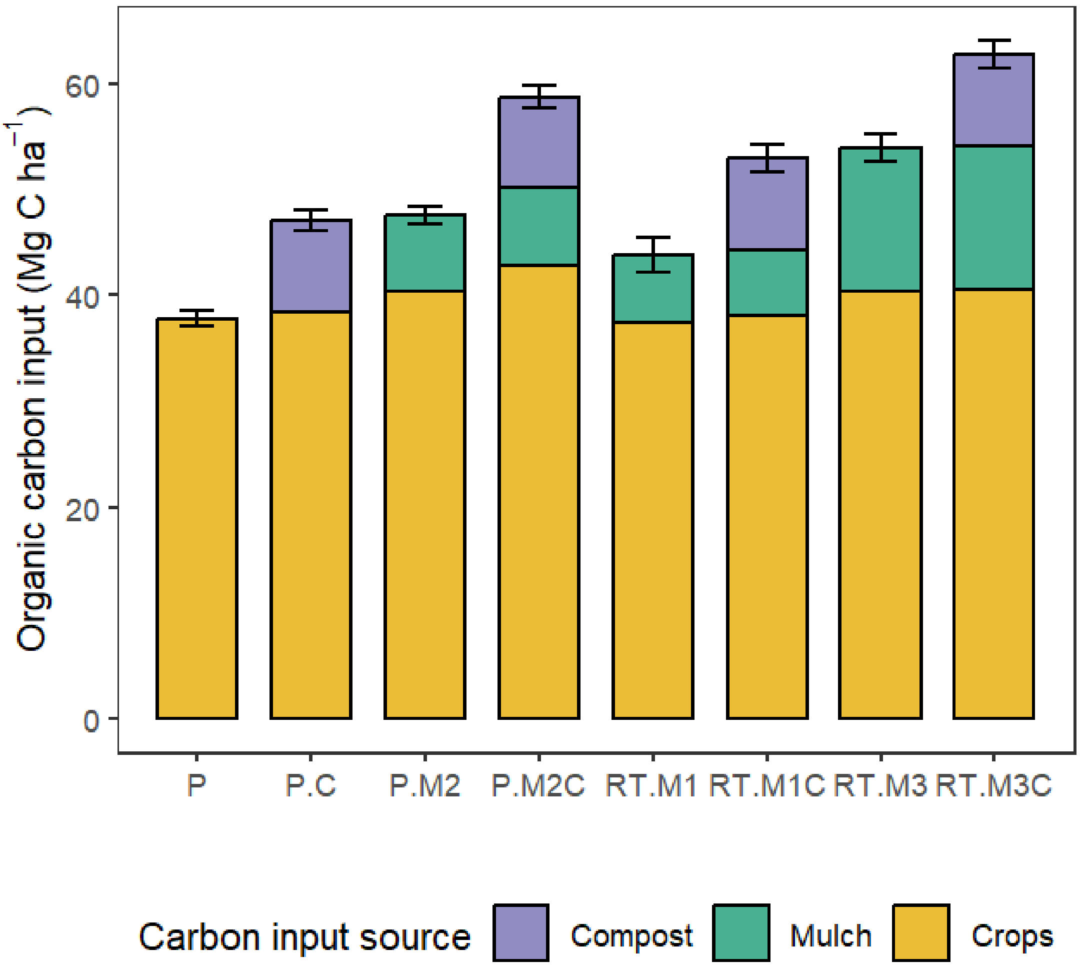

Figure S2 Organic carbon input over the experimental course of ten years from three C input sources (violet: compost, green: mulch, yellow: crops) in the eight treatments (P: plough; C: compost; M: mulch, where M1 and M2 refer to the first and second mulching, respectively, M3 to the double mulched treatments; RT: reduced tillage, and their combinations). "Crops" include all organic C input from vegetation that remains in the field.

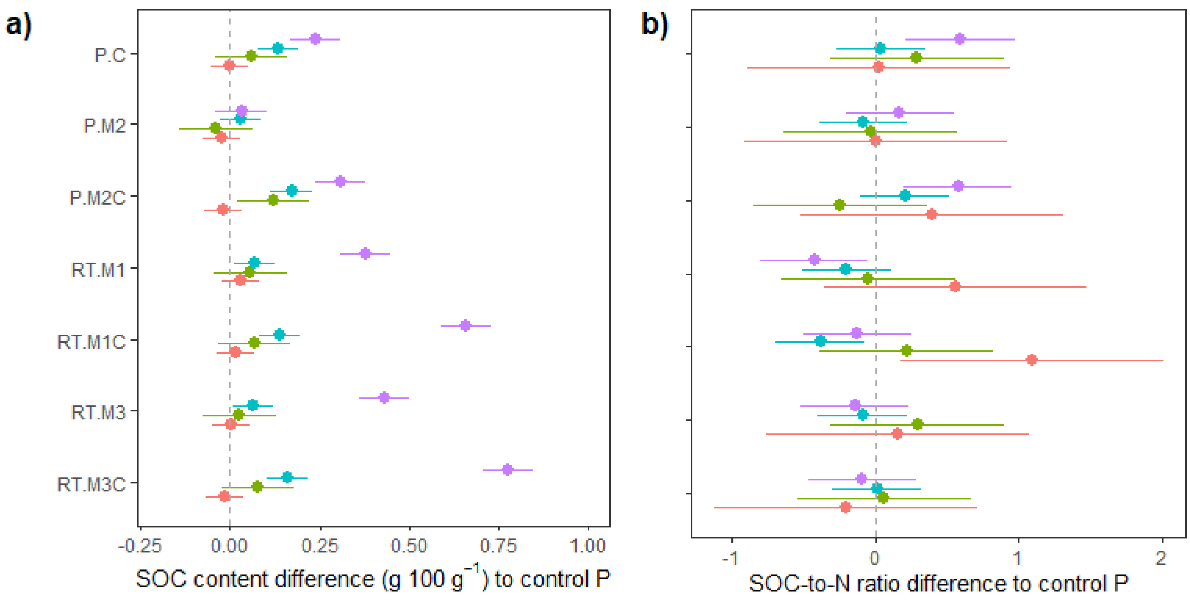

Figure S3 Difference (estimates and 95% confidence intervals) of a) soil organic carbon (SOC) content (%) and b) SOC-to-N ratio separated for soil layers (indicated by colours) of the treatments with one, two or three regenerative measures (C: compost; M: mulch, where M1 and M2 refer to the first and second mulching, respectively, M3 to the double mulched treatments; RT: reduced tillage, and their combinations) to the control (P: plough). The difference from the control is significant when the confidence intervals do not cross the dashed line.
